# Effectiveness and safety of prolonged prone positioning in adult patients with acute respiratory distress syndrome (ARDS): a systematic review and meta-analysis

**DOI:** 10.1186/s13054-025-05712-0

**Published:** 2025-11-06

**Authors:** Carolin Jung, Hans-Joerg Gillmann, Thomas Stueber

**Affiliations:** Department of Anaesthesiology and Intensive Care Medicine, Hannover Medical School, Carl-Neuberg-Strasse 1, 30625 Hannover, Germany

**Keywords:** Prolonged prone, ARDS, Meta-analysis, Systematic review

## Abstract

**Background:**

Prolonged prone positioning (PPP) for ≥ 24 h may enhance outcomes in moderate to severe acute respiratory distress syndrome (ARDS), but may also increase risks such as pressure injuries and complications. Despite clinical rationale, high-quality evidence for PPP’s safety and efficacy remains scarce.

**Methods:**

We conducted a systematic review and meta-analysis of randomized controlled trials (RCT) and observational studies. Trials that compared two distinct treatment groups in adult patients with ARDS were included: prone position < 24 h (standard) and ≥ 24 h (prolonged). Databases searched included MEDLINE, CENTRAL, ClinicalTrials.gov, ISRCTN, ICTRP and the Cochrane Covid-19 Study Register (last search: 3 July 2025). Risk of bias was assessed using ROB-2 for RCTs, and the ROBINS-I V2 tool for non-randomised intervention studies (NRSI). The primary outcome was mortality. Secondary outcomes included improvement of oxygenation and adverse events. Outcomes (Risk ratios and hazard ratios) were calculated using a random-effect model with 95% confidence intervals (CI). The quality of evidence was evaluated using the GRADE assessment.

**Results:**

Of 19,986 records, 9 (*n* = 1,045) were included in the qualitative and quantitative analysis. Four studies, including two small RCTs (*n* = 112) and two NRSIs (*n* = 581), had a low to moderate risk of bias. Most studies included patients with COVID-19 ARDS. Meta-analysis showed no significant effect on 90-day mortality (*n* = 641, HR 0.72; 95% CI 0.41–1.25). No heterogeneity was detected among studies (I² = 0%), but the confidence interval for I² was wide (95% CI: 0–89%), suggesting the possibility that substantial heterogeneity may exist. Similarly, no significant differences were found for secondary outcomes.

**Discussion:**

Current evidence does not support the use of PPP outside of clinical studies. Pooled data from small trials and NRSIs reveal no significant effect of PPP on mortality, oxygenation, or safety outcomes. The evidence is of low to very low certainty, limited by inconsistency and imprecision. The wide confidence intervals indicate low statistical power, therefore both harm and benefit remain plausible on the basis of the available evidence. Well-powered RCTs are needed to clarify the potential benefits and risks of PPP in ARDS.

**Supplementary Information:**

The online version contains supplementary material available at 10.1186/s13054-025-05712-0.

## Background

Prone positioning (PP) is a well-established therapeutic strategy in the management of moderate to severe acute respiratory distress syndrome (ARDS). Its physiological rationale lies in promoting a more homogeneous distribution of lung stress and strain [1]. By redistributing transpulmonary pressures, PP facilitates alveolar recruitment, reduces regional overdistension, and mitigates cyclic alveolar collapse - all of which contribute to minimizing ventilator-induced lung injury (VILI). Moreover, PP improves ventilation-perfusion matching, frequently resulting in enhanced gas exchange. Recent evidence suggests a potential dose response relationship, with clinical benefits such as improved oxygenation and reduced mortality being more pronounced when PP is initiated early and maintained for extended durations [2, 3]. Randomized trials and prior meta-analyses support the efficacy of daily prone sessions lasting at least 16 h in improving survival among patients with moderate to severe ARDS [4]. Theoretical and physiological considerations further propose that extending proning sessions beyond 24 h may offer additional benefits, including sustained improvements in lung mechanics and gas exchange, fewer position-related interruptions, and more consistent prevention of VILI [5]. However, prolonged prone positioning may also be associated with an increased risk of certain complications. These include a higher incidence of pressure injuries, such as skin and soft tissue damage, particularly at bony prominences, and a greater need for sedation, which may contribute to longer durations of mechanical ventilation and intensive care unit (ICU) stay [5]. There may also be an increased risk of accidental extubation or displacement of vascular lines during repositioning maneuvers.

Despite growing clinical interest, data on the efficacy and safety of prolonged prone positioning (PPP) remain limited. To address this knowledge gap, we conducted a systematic review and meta-analysis to assess the impact of PPP on relevant clinical outcomes in patients with ARDS.

## Methods

We conducted this study in accordance with the Preferred Reporting Items for Systematic Reviews and Meta-Analysis (PRISMA) guidelines and the Cochrane Handbook for Systematic Reviews of Intervention. The study was registered at the International prospective register of systematic reviews (PROSPERO) (CRD42023413572).

### Eligibility criteria

The inclusion criteria were as follows: [1] original research articles, including randomized controlled trials as well as retrospective or prospective observational studies; [2] published in either English or German; [3] specifying a clearly defined duration of prone positioning; [4] comparing patients receiving standard prone positioning therapy (< 24 h) with those undergoing prolonged prone positioning (≥ 24 h); [5] involving adult patients; [6] diagnosed with ARDS; and [7] reporting relevant outcomes of interest. The exclusion criteria were as follows: [1] case reports and case series with fewer than five patients [2], studies involving non-human subjects.

Studies were grouped for synthesis according to intervention type, specifically comparing prolonged versus standard duration of prone positioning, as well as by outcome measures such as short- and long-term mortality, total duration of prone position, adverse effects, and change in oxygenation while in prone position. The rationale for these groupings was based on clinical relevance and methodological consistency across studies. Initially, we intended to perform separate analyses for randomized controlled trials (RCT) and non-randomized studies of interventions (NRSI) in accordance with our review protocol. However, due to the limited quality and small sample sizes of the available RCTs, which consisted primarily of underpowered pilot studies, and the presence of NRSIs with adequate adjustment for confounders and an overall low risk of bias, we opted to pool data from both study designs. This approach allowed for a more comprehensive synthesis of the available evidence while maintaining methodological rigor.

### Information sources

Literature searches were systematically conducted in the following databases: MEDLINE, CENTRAL, ClinicalTrials.gov, the ISRCTN registry (http://isrctn.com), the World Health Organization International Clinical Trials Registry Platform (ICTRP) (www.who.int/clinical-trials-registry-platform), and the Cochrane COVID-19 Study Register (https://covid-19.cochrane.org). The final search for each database was completed on 3 July 2025.

### Search strategy

The full search strategy is provided in the Supplement.

### Selection process

The screening and selection process was conducted using the Systematic Review Management Platform Rayyan [6]. Duplicates were removed manually by CJ using Rayyan’s duplication identification strategy. After deduplication, initial title and abstract screening were performed by two independent reviewers (CJ, TS). Full text for titles that passed screening were retrieved and screened for eligibility according to the predefined inclusion and exclusion criteria. Disagreements were resolved by consensus or consultation with a third reviewer (HJG).

### Data collection process

Data were independently extracted by two reviewers (CJ and TS) using a standardized data extraction form (Microsoft Excel, Redmond, WA: Microsoft Corporation; 2024), capturing study characteristics, patient demographics, and intervention details. All data was extracted from published reports. We did not seek supplementary data beyond what was publicly available in the published articles.

### Data items

Data extracted included study design (RCT or NRSI), study setting (single- or multi-center), geographic region, sample size, and, for NRSIs, the method of adjustment for baseline confounding. Patient characteristics comprised age, body mass index (BMI), severity of ARDS, and the PaO₂/FiO₂ ratio before the first prone session. Intervention variables included duration of the first prone session, average session duration, cumulative prone time, number of position changes, use of neuromuscular blockade, use of adjunctive ECMO therapy, and specification of predefined criteria for discontinuation of prone positioning. Primary outcomes comprised short-term mortality (10–30 days or ICU mortality) and long-term mortality (> 30 days), chosen for their clinical relevance and consistent reporting. Secondary outcomes comprised improvement in gas exchange, cumulative prone time, number of sessions, ICU length of stay, duration of mechanical ventilation, and adverse events (e.g., pressure ulcers, accidental extubation, displacement of tubes/catheters, and changes in respiratory mechanics). For missing or unclear information, we inferred values from context, documenting such assumptions and accounting for them in risk-of-bias and certainty assessments. A detailed descriptions of all such assumptions is provided in the Supplement. Owing to substantial heterogeneity among eligible studies and limited data availability for several predefined endpoints specified in the study protocol, we were unable to collect sufficient data on these outcomes to permit quantitative synthesis. Specifically, only a small number of studies reported on changes in pulmonary carbon dioxide clearance, length of ICU and hospital stay, duration of mechanical ventilation, chest tube displacement, liver dysfunction, acute kidney injury, cardiovascular instability, ocular complications, ventilation distribution, and respiratory mechanics. Additionally, while the initial review protocol planned to compare a standard prone positioning group (≤ 24 h per session) with a prolonged prone positioning group (> 24 h per session), the majority of included studies utilized a cutoff of < 24 h versus ≥ 24 h per session. Accordingly, we adapted our analyses to ensure methodological consistency across comparisons.

### Study risk of bias assessment

Risk of bias for each primary endpoint was independently assessed by two reviewers (CJ, TS). The Cochrane Risk of Bias 2 (ROB2) tool was used for randomized trials, and the Risk Of Bias In Non-randomised Studies of Interventions (ROBINS-I V2) tool for observational studies. Overall risk of bias was considered high or critical if any domain was rated as high or critical.

### Effect measures

For the meta-analyses examining short and long-term mortality, effect estimates as reported in the original studies were used. Because of the small number of available RCTs, RCTs and NRSIs were pooled for the main analyses. For each endpoint, we conducted two sets of analyses: (i) including all eligible studies, and (ii) restricted to studies with low to moderate risk of bias. In addition, several sensitivity analyses were performed, separating RCTs from observational studies.

For the analysis of short-term mortality including all eligible studies, risk ratios were calculated from raw event counts, as time-to-event data were not available for most studies. For the analysis of short-term mortality restricted to studies with low to moderate risk of bias, only RCT data could be included. The sole eligible non-randomized study employed an inverse probability treatment weighting (IPTW) design, which precluded a direct comparison of raw event counts without substantially increasing the risk of bias to a critical level due to loss of adjustment. As the RCTs also lacked time-to-event data, hazard ratios could not be derived. Accordingly, the short-term mortality analysis was based exclusively on RCT data. Long-term mortality data were available exclusively from RCTs and NRSIs assessed as having low to moderate risk of bias. These datasets were pooled for meta-analysis, and adjusted hazard ratios (aHRs) were extracted from NRSIs to improve comparability and account for baseline differences. HR were log-transformed prior to quantitative synthesis. In one RCT [7], HRs had to be approximated from the Kaplan–Meier curve. When studies reported medians and interquartile ranges instead of means and standard deviations, these were estimated using the Box-Cox method and subsequently used for quantitative synthesis.

For the meta-analyses of total prone time and changes in PaO₂/FiO₂, standardized mean difference (SMD) was used as an effect estimate. For the meta-analyses of pressure ulcers and accidental loss of catheters or endotracheal tubes, data from both RCTs and NRSIs were pooled to capture the broadest possible range of reported complications. For both outcomes, RR were calculated directly from the observed raw event counts without adjustment for potential confounders.

### Synthesis methods

Eligibility for each synthesis was determined by comparing the intervention characteristics, summarized in the “Characteristics of Included Studies” table, against the predefined criteria for each group. All statistical analyses were performed in R Statistical Software (R Foundation for Statistical Computing, Vienna, Austria, Version 4.4.1), utilizing the ‘meta’ package for data synthesis. We calculated pooled estimates and their corresponding confidence intervals using a random-effects model with the inverse variance method and the Hartung-Knapp adjustment, as the number of included studies was low and substantial heterogeneity existed between studies. The between-study variance (τ²) was estimated using the Paule-Mandel method, and its confidence interval, was obtained via the Q-profile method. We calculated I² with its corresponding confidence interval due to the low number of studies included in the meta-analysis, as I² can be biased when the number of included studies is small [8].

### Reporting bias assessment

Publication bias was assessed using the ROB-ME tool [9]. The evaluation indicated a low risk of publication bias across the included studies. This suggests that the results of our systematic review and meta-analysis are unlikely to be substantially influenced by selective reporting or non-publication of relevant studies. Funnel plots were not constructed due to the inclusion of fewer than ten studies in each meta-analysis.

### Certainty assessment

We assessed the certainty of the evidence using the Grading of Recommendations Assessment, Development, and Evaluation (GRADE) framework [10, 11], which systematically evaluates multiple domains, including risk of bias, indirectness, imprecision, inconsistency, and the possibility of publication bias. The certainty of evidence was graded separately for each patient-important outcome, with ratings determined independently by two authors (CJ, TS). Any disagreements were resolved by consensus. Summary of Findings tables were prepared using GRADEpro GDT Software [11]. We restricted our certainty assessment to studies at low or moderate risk of bias, consistent with GRADE recommendations to minimize bias and enhance the reliability of evidence synthesis [12].

## Results

### Study selection

The systematic search identified 19,986 records. After removing 4,774 duplicates, 15,212 records remained for title and abstract screening, of which 15,184 were excluded based on predefined eligibility criteria. Full texts of 28 articles were assessed, leading to the exclusion of:

- 16 studies that did not compare prolonged (≥ 24 h) with standard (< 24 h) prone positioning [13–28].
- Two studies without definitive prone session duration [29, 30].
- One study that was a case series with < 5 cases per arm [31].

A detailed summary of the characteristics of the excluded studies is provided in Table S1.

**Fig. 1:**
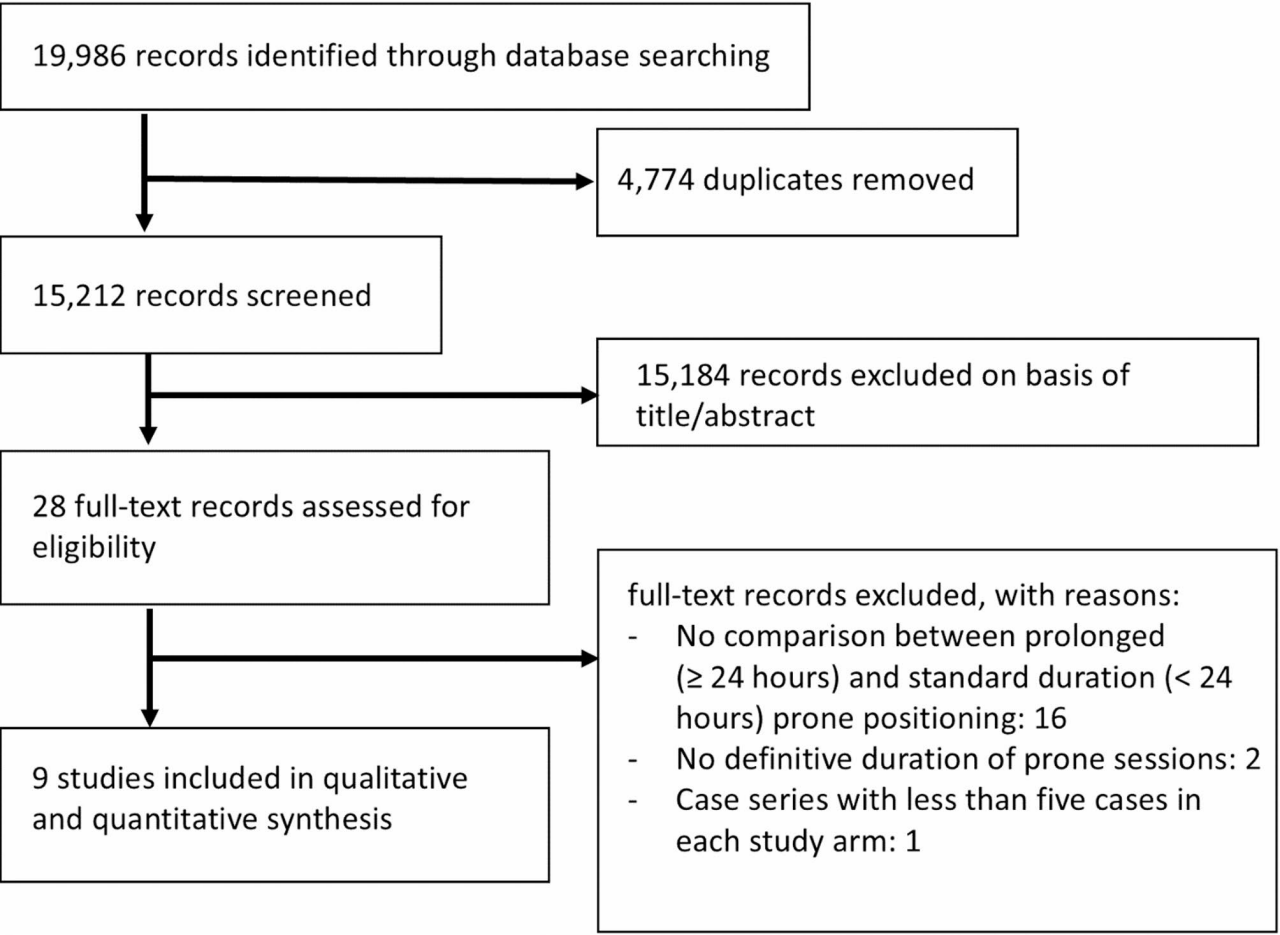
Study selection process diagram

Following full-text screening, 9 studies were included in the synthesis [7, 32–39] (Table 1). The study selection process is illustrated in Fig. 1.

**Table 1:** Characteristics of included studies

Study	Publication date	Design	Study setting	Region	Population	Baseline PaO₂/FiO₂	Age	BMI	Duration of PP /prolonged	Duration of PP /standard	Case number	Outcomes
Rezoagli et al.	2021	Retrospective NRSI	Single-center	Italy	Moderate-to-severe ARDS, COVID-19	102 ± 43 vs. 119 ± 49	59 ± 11 vs. 65 ± 7	29*	39 ± 6	17 ± 3	38 (15 vs. 23)	ICU mortality; Change in PaO₂/FiO₂ ratio, Change in PaCO_2_, Lung mechanics (∆P; *C*_*rs*_; Standardized minute ventilation), pressure injuries, ICU LOS
Shinner et al.	2021	Retrospective NRSI	Single-center	United Kingdom	NA	NA	NA	NA	73.6 (4 - 108) hours	18.2 (1 - 24) hours	103 (43 vs. 60)	Mortality (without further specification), pressure injuries
Lucchini et al.	2021	Retrospective NRSI	Single-center	Italy	Moderate-to-severe ARDS, COVID-19	111 (71–145) vs. 120 (86–150)	58 (55–66) vs. 60 (53–68)	28 (26–33) vs. 28 (26–33)	34 (30–41)	16 (15–18)	96 (37 vs. 59)	Pressure injuries; Total duration of proning
Hafez et al.	2022	RCT	Single-center	Egypt	Severe ARDS, COVID-19	79 ± 31 vs. 84 ± 31	51 ± 7 vs. 48 ± 10	31 ± 4 vs. 31 ± 4	24**	16**	52 (26 vs. 26)	28-day-Mortality, Change in PaO₂/FiO₂ ratio, Lung mechanics (*C*_*rs*_). pressure injuries, nerve injuries
Page et al.	2022	RCT	Single-center	USA	Moderate-to-severe ARDS, COVID-19	99 ± 27 vs. 93 ± 25	64 ± 14 vs. 62 ± 12	35 ± 9 vs. 32 ± 6	23 ± 6	15 ± 4	52 (26 vs. 26)	30 day-Mortality, Change in PaO₂/FiO₂ ratio; Lung mechanics (∆P; *C*_*rs*_); pressure injuries; loss-of-catheters or -endotracheal tube: Total duration of proning
Karlis et al.	2023	Retrospective NRSI	Single-center	Greece	Moderate-to-severe ARDS, COVID-19	97.6 (27.4) vs. 103.4 (25.8)	61.5 (15.1) vs. 66.5 (9.7)	33 ± 7 vs. 31 ± 5.5)	46 (40 - 48)	20 (20 −22)	63 (37 vs. 26)	28-day-mortality; Change in PaO₂/FiO₂ ratio, Change in PaCO_2_, Lung mechanics (P_plat_, ∆P, *C*_*rs*_;); number of prone sessions, Total duration of proning
Okin et al.	2023	Retrospective NRSI; Adjustment for baseline confounding (IPTW)	Multi-center	USA	Moderate-to-severe ARDS, COVID-19	158 (105 - 211) vs. 149 (99 - 211)	63 (52–70) vs. 60 (51–74)	30 (27–35) vs. 30 (26–36)	40 (27–55)***	17 (14–20)***	267 (157 vs. 110)	30-day and 90-day mortality; Change in PaO2/FIO2 ratio; Lung mechanics (∆P; *C*_*rs*_; ventilatory ratio); adverse events (Arrhythmias, hypotension, Loss-of-catheters or endotracheal tube; Pressure injuries; rhabdomyolysis); hospital and ICU LOS; Total duration of proning
Hochberg et al.	2024	Retrospective NRSI; Adjustement for baseline confounding (IPTW)	Multi-center	USA	Moderate-to-severe ARDS, COVID-19	76 (65–96) vs. 82 (68–96)	60 (52–69) vs. 63 (48–73)	33 (28–40) vs. 32 (26–37)	45 (34–64)***	19 (17–20)***	314 (234 vs. 80)	90-day mortality; Change in PaO₂/FiO₂ ratio; Total duration of proning
Saez de la Fuente et al.	2024	RCT	Single-center	Spain	Moderate-to-severe ARDS, COVID-19	137 ± 64 vs. 119 ± 41	58 (46–65) vs. 50 (36–57)	34 ± 9 vs. 32 ± 6	48**	16**	60 (30 vs. 30)	90 day- and 28-day mortality; Change in PaO₂/FiO₂ ratio; Change in PaCO_2_; lung mechanics (P_plat_, ∆P, *C*_*rs*_; ventilatory ratio; mechanical power); Hospital and ICU LOS; Total duration of proning; adverse events (loss-of-catheters and endotracheal tube; pressure injuries)

Data are presented as mean ± standard deviation or median (Q1–Q3). When two groups are compared (vs.), values for the prolonged group are listed first, followed by those for the standard group. *approximated by mean weight + height; **according to study protocol; ***first session

*C*_*rs*_: *static compliance of the respiratory system; ICU: intensive care unit; LOS: length of stay; ∆P: Driving pressure; NRSI: non-randomized study of interventions; PaO₂/FiO₂ ratio: ratio of arterial partial pressure of oxygen to fractional inspired oxygen; P*_*plat*_: *Plateau pressure; RCT: randomized controlled trial*

### Study characteristics

Nine studies met the inclusion criteria: three randomized controlled trials (RCTs) and six non-randomized studies, two of which adjusted for baseline confounding. Eight studies focused exclusively on COVID-19–related ARDS [7, 32–36, 38, 39], while one [37] did not specify the ARDS etiology. Across studies, mean participant age ranged from 48 to 68 years. The baseline PaO₂/FiO₂ ratio varied between 76 and 159; in four studies [32, 33, 35, 36] it was < 100, indicating more severe hypoxemia. Mean body mass index (BMI) was 28–35 in all but one study [37], which did not report BMI.

Prone positioning duration in intervention groups varied markedly: ~24 h in two RCTs [32, 33], 34 h in one study [39], 40–48 h in five studies [7, 34–36, 38], and a median of 74 h in one study [37]. In control groups, durations averaged 15–20 h.

### Risk of bias in studies

Among RCTs, the overall risk of bias was low in one study [32], some concerns in one [7], and high in one [33] (Table 2). For NRSI, risk of bias was low in two studies with adequate adjustment for baseline confounding [34, 35], and critical in the remaining studies [36–39] (Table 2). Key concerns included selective reporting in RCTs [7, 33], and absence of baseline confounding adjustment in most NRSI. One RCT [33], was judged as having a high risk of bias (Table S6C), primarily due to potential deviations from intended interventions and missing outcome data.

**Table 2:** Risk-of-bias assessment for RCTs and NRSI

Risk-of-bias assessment: RCT											
	**Bias arising from randomization process**	**Bias due to deviations from intended interventions**		**Bias due to outcome data**		**Bias due to missing outcome data**		**Bias in selection of reported results**		**Overall**	
**Saez de la Fuente**,** 2024**	Low	Low		Low		Low		Some concerns		Some concerns	
**Page**,** 2022**	Low	Low		Low		Low		Low		Low	
**Hafez**,** 2022**	Some concerns	High		High		Low		Some concerns		High	

**Risk-of-bias assessment: NRSI**											
	**Control for confounding?**	**Potential for confounding?**	**Outcome measurement inappropriate?**	**Bias due to confounding**	**Bias due to selection of participants**	**Bias in classification of interventions**	**Bias due to deviations from intended interventions**	**Bias due to missing data**	**Bias in measurement of outcomes**	**Bias in reported result**	**Overall**
**Hochberg 2024**	Yes	Yes	No	Low	Low	Low	Low	Low	Low	Low	Low
**Okin, 2023**	Yes	Yes	No	Low	Low	Low	Low	Low	Low	Low	Low
**Karlis, 2023**	No	Yes	No	-	-	-	-	-	-	-	Critical
**Rezoagli**,** 2021**	No	Yes	No	-	-	-	-	-	-	-	Critical
**Shinner**,** 2021**	No	Yes	No	-	-	-	-	-	-	-	Critical
**Lucchini**,** 2021**	No	Yes	No	-	-	-	-	-	-	-	Critical

“-“: No information is provided, as all studies were deemed to be at critical risk of bias during Step B: Preliminary Considerations of the ROBINS-I V2 tool, rendering further detailed risk-of-bias assessment unnecessary

*NRSI: non-randomized study of interventions; RCT: randomized controlled trial “-“*

### Primary outcome

Across the included studies, mortality was assessed at varying time points. Three studies reported 28‑day mortality [7, 33, 36], two reported 30‑day mortality [32, 34], and three reported 90‑day mortality [7, 34, 35]. One study assessed ICU mortality [38], while in another the exact timeframe for mortality assessment was not specified [37].

**Fig. 2:**
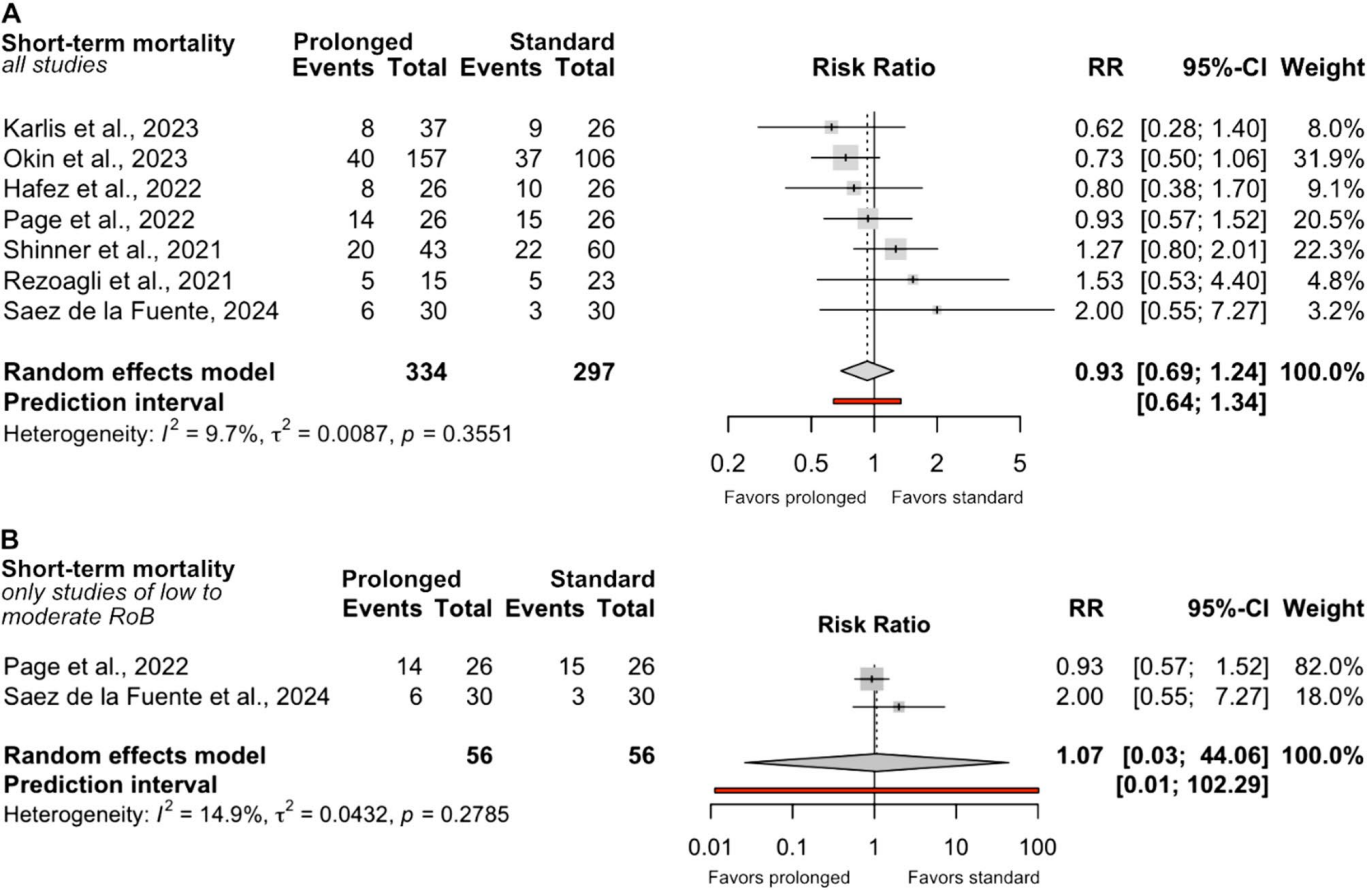
Comparison of short–term mortality between prolonged and standard prone position durations. **A** = Synthesis including all studies reporting short-term mortality. **B** = Synthesis restricted to studies with low to moderate risk of bias

In a pooled analysis of all studies reporting short-term mortality (*n* = 631), no significant difference was observed between standard and prolonged prone positioning (RR 0.93; 95% CI 0.69–1.24; Fig. 2A). In an analysis restricted to studies with low or moderate risk of bias, two small randomized controlled trials (112 patients in total) were included. The pooled analysis showed no significant difference between standard and prolonged prone positioning (RR 1.07; 95% CI 0.03–44.04; I² = 14.9%; Fig. 2), with the very wide confidence interval indicating substantial imprecision allowing for both meaningful harm and benefit.

**Fig. 3:**
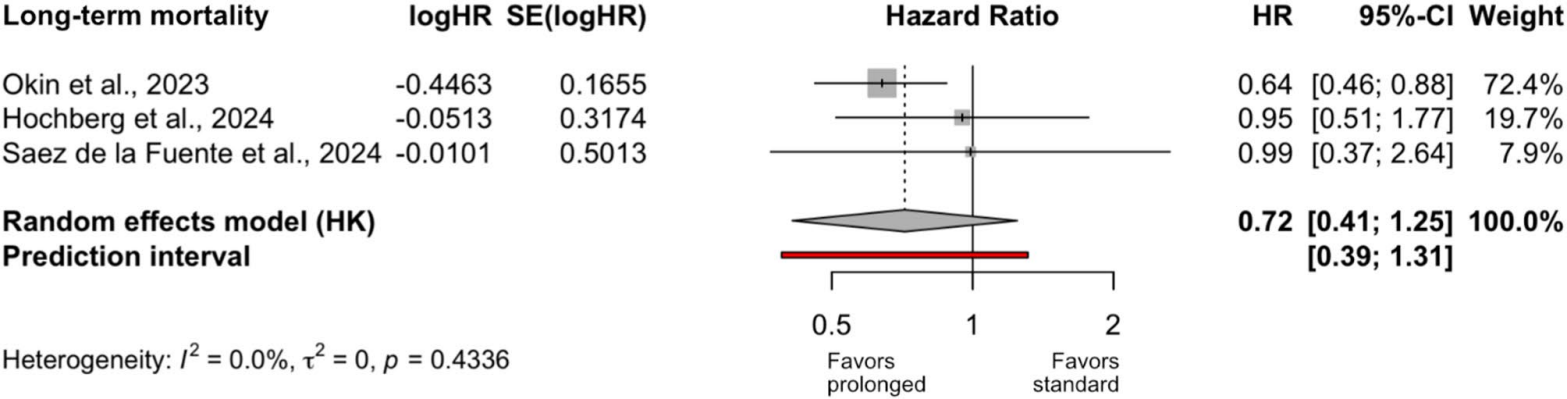
Comparison of long-term mortality between prolonged and standard prone position durations. All included studies were of low to moderate risk of bias

Three studies (two NRSIs and one RCT; 641 patients) reported 90‑day mortality, all of which were assessed as having low or moderate risk of bias. Meta-analysis again showed no significant effect of prolonged vs. standard prone duration (aHR 0.72; 95% CI 0.41–1.25; Fig. 3). While the point estimate favored prolonged prone positioning this trend was largely driven by the Okin study [34], which exerted high influence due to its narrow confidence interval and comparatively larger sample size. The meta-analysis revealed no observed heterogeneity (I² = 0%). However, the 95% confidence interval for I² was wide (0–89%), indicating considerable uncertainty regarding the true degree of between-study heterogeneity. This suggests that, although no heterogeneity was detected, the presence of substantial heterogeneity cannot be excluded with confidence, potentially due to limited power or a small number of studies.

### Secondary outcomes

**Fig. 4:**
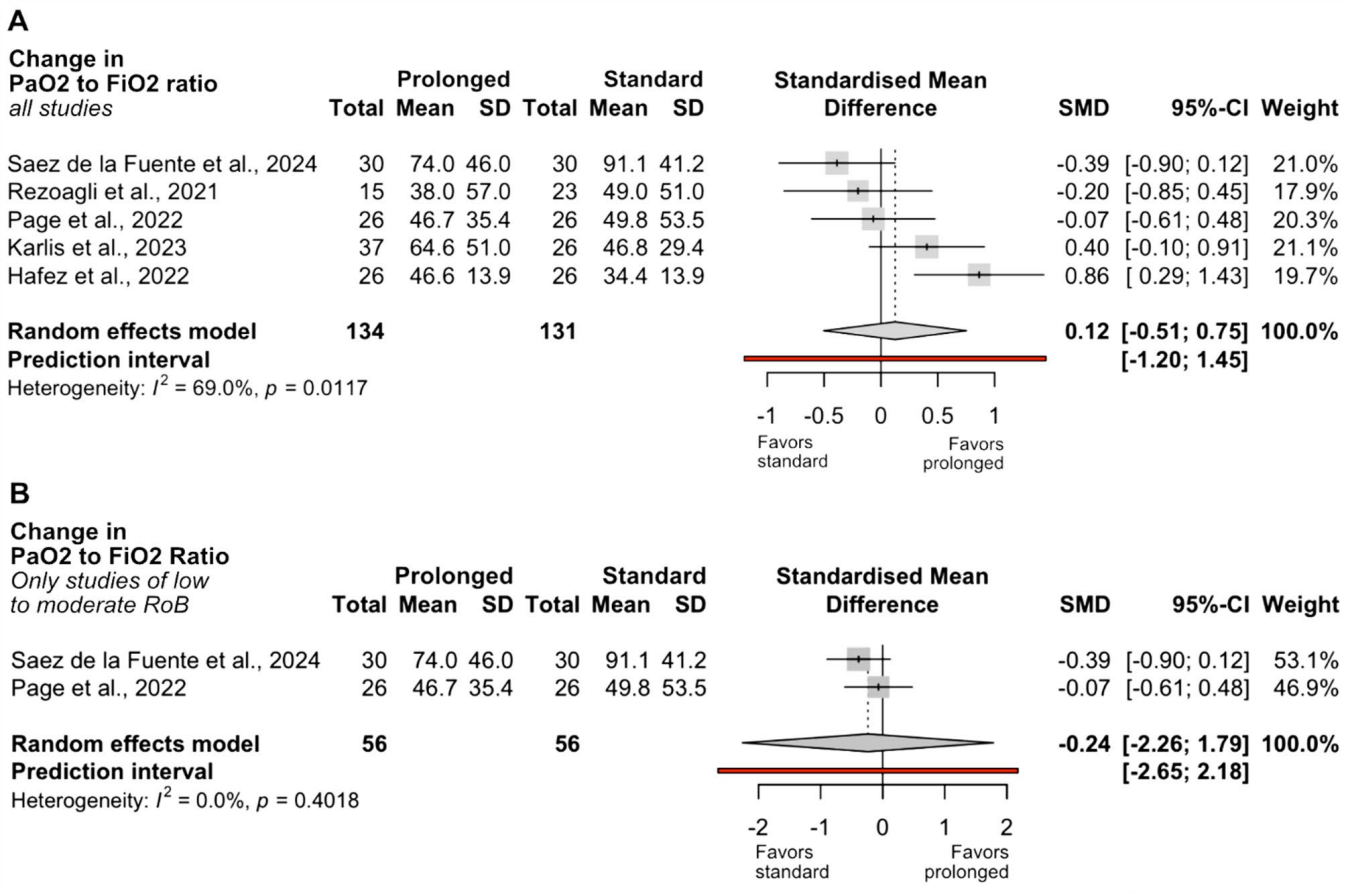
Comparison of change in PaO₂/FiO₂ ratio in prone position between prolonged and standard prone groups. **A** = Synthesis including all studies reporting change in PaO₂/FiO₂ ratio. **B** = Synthesis restricted to studies with low to moderate risk of bias

Considering all available studies reporting oxygenation changes during prone positioning, no significant difference was observed in a pooled analysis of three small RCTs and two NRSI without adjustment for baseline confounding (SMD 0.12; 95% CI −0.51 to 0.75; I² = 69%; *n* = 265; Fig. 4A). In the analysis restricted to studies with low to moderate risk of bias, considerable heterogeneity in the timing of arterial blood gas measurements following initiation of prone positioning limited the analysis of oxygenation changes to two small RCTs. Notably, the larger NRSIs with adequate baseline adjustment reported PaO₂/FiO₂ ratios only up to 16 h into the first prone session, providing no data on the effects of prolonged prone positioning. The meta-analysis of the available RCT data found no significant difference in oxygenation change between prolonged and standard prone durations (SMD − 0.24; 95% CI − 2.26 to 1.79; I² = 0%; *n* = 112; Fig. 4B).

**Fig. 5:**
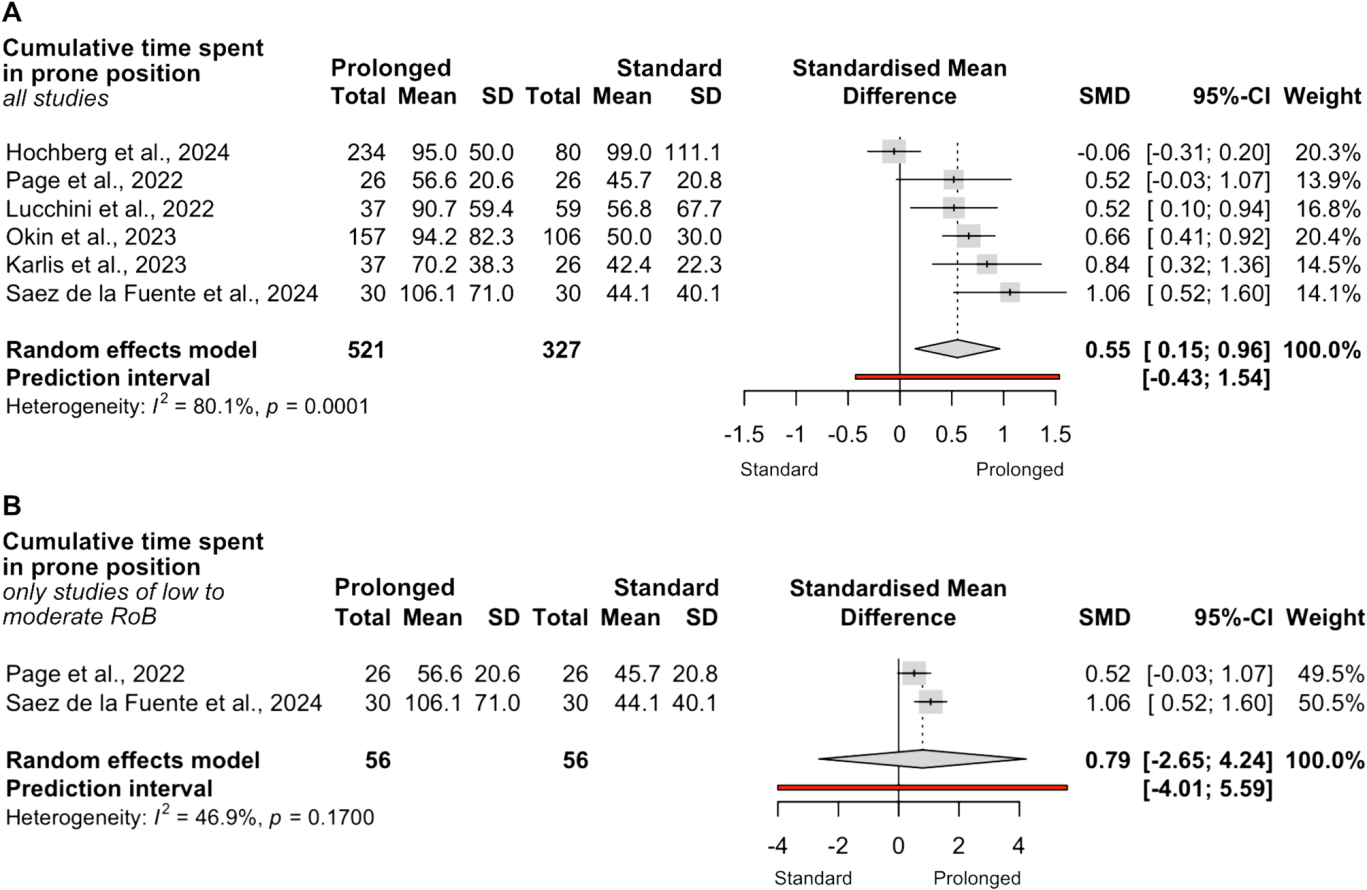
Comparison of cumulative time spent in prone position between prolonged and standard prone groups. **A** = Synthesis including all studies reporting cumulative time spent in prone position. **B** = Synthesis restricted to studies with low to moderate risk of bias

Six studies reported cumulative time spent in prone position (*n* = 848). The comprehensive analysis suggested a higher cumulative exposure in the prolonged-prone group (SMD 0.55; 95% CI 0.15 to 1.6; I² = 80.1%; Fig. 5A), although it was largely based on unadjusted non-randomized data. When restricted to studies with low to moderate risk of bias, two RCTs (*n* = 112) reported cumulative time spent in prone position. None of the NRSIs reported adjusted data for this endpoint, and therefore none could be included in the restricted analysis. In this analysis, no significant difference was observed between groups (SMD 0.79; 95% CI − 2.65 to 4.24; I² = 46.9%; Fig. 5B). Heterogeneity was considerable, reflecting large differences in reported cumulative durations across studies.

### Adverse events

**Fig. 6:**
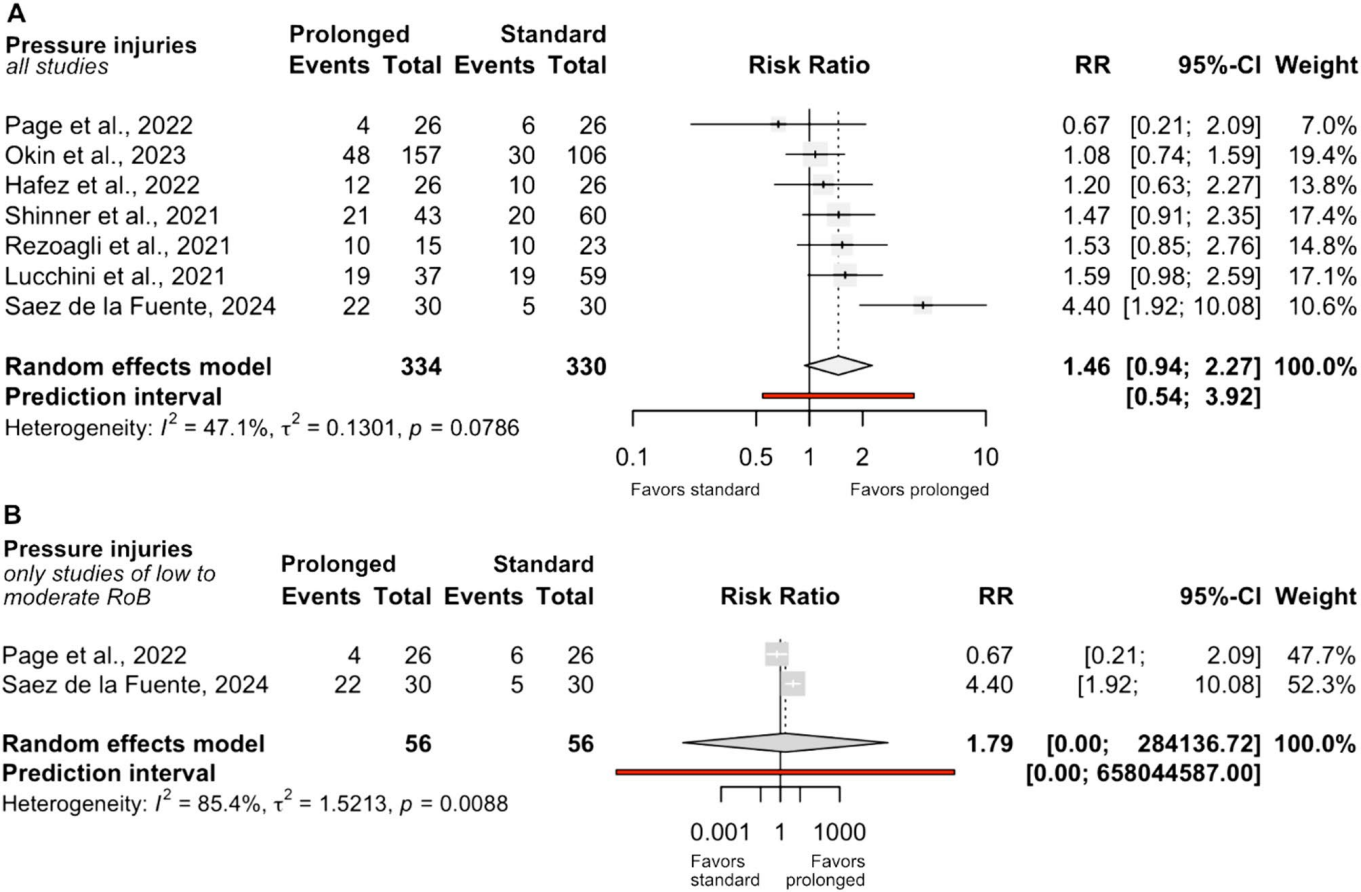
Comparison of the incidence of pressure injuries between prolonged and standard prone position groups. **A** = Synthesis including all studies reporting the incidence of pressure injuries. **B** = Synthesis restricted to studies with low to moderate risk of bias

Overall, seven studies reported the incidence of pressure injuries. In the crude analysis including all results, no significant difference was observed between groups (RR 1.46; 95% CI 0.94 to 2.27; I² = 47.1%; *n* = 664; Fig. 6A). When restricted to studies with low to moderate risk of bias, two RCTs studies (*n* = 112) were available. None of the NRSIs reported adjusted data for this endpoint. Meta‑analysis again revealed no significant difference between groups (RR 1.79; 95% CI 0.00–284,136.72; I² = 85.4%; Fig. 6B), although heterogeneity was substantial, largely reflecting variability in the frequency of reported injuries. The trial by Sáez de la Fuente et al. [7] showed the highest risk in the prolonged‑prone group, corresponding with both the longest cumulative prone duration and the largest difference between intervention and control groups (Fig. 4).

**Fig. 7:**
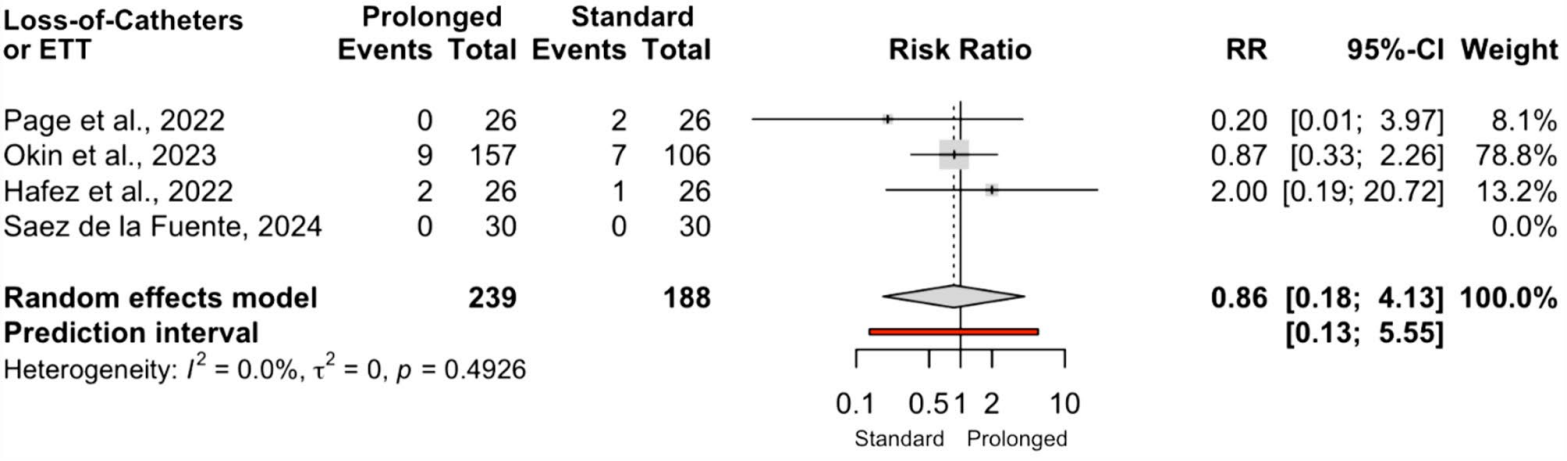
Comparison of the incidence of loss-of-catheters or endotracheal tubes between prolonged and standard prone position groups. Synthesis includes all studies reporting the incidence of loss-of-catheters or endotracheal tubes

Four studies (three RCTs and one NRSI; 427 patients) reported the incidence of catheter or endotracheal tube dislodgement. These events were rare across all studies, with no heterogeneity detected (I² = 0%). Meta‑analysis demonstrated no significant difference between prolonged and standard pronation (RR 0.86; 95% CI 0.18–4.13; Fig.  7).

### Sensitivity analyses

Sensitivity analyses including (a) separate analyses of RCT and NRSI data and (b) mortality analysis at the latest available timepoint, including each study’s maximum reported follow-up (e.g., 30-day, 90-day, or ICU mortality), did not reveal any trends or statistically significant associations beyond those observed in the main analyses (Figures S1–S2).

### Reporting bias

Publication bias was assessed using the ROB-ME tool (Table S5). Across all conducted meta-analyses, the evaluation indicated a low risk of publication bias. This suggests that the available evidence is unlikely to be substantially influenced by selective publication of studies, thereby increasing confidence in the overall validity and robustness of the synthesized findings.

### Certainty of evidence

The certainty of the evidence from studies at low to moderate risk of bias concerning both the primary outcomes and secondary endpoints is rated from low to very low (Table S3). This downgrading primarily stems from serious to extreme imprecision, as well as serious to very serious inconsistency.

### Ongoing studies

At present, three randomized controlled trials are being conducted to assess the efficacy of prolonged prone positioning (Table S2).

## Discussion

Current evidence does not support the use of prolonged prone positioning outside of clinical trials. To date, no adequately powered RCT has specifically addressed this question. Our systematic review identified three RCTs and six observational studies comparing prolonged with standard prone positioning. One RCT [33] and four observational studies [36–39] were of high or critical risk of bias, leaving limited high-quality data for pooled analysis. None of the analyses, including those with all eligible studies, those restricted to low-to-moderate risk of bias studies, and sensitivity analyses, revealed statistically significant effects of PPP on primary or secondary outcomes assessing effectiveness or adverse events (Table 3). The wide confidence intervals observed throughout our analysis reflect limited statistical power. Consequently, neither harm nor benefit can be definitely excluded, which underscores the uncertainty of these findings. Most studies enrolled patients with COVID‑19–related ARDS, limiting the generalizability of these findings to other ARDS etiologies. Notably, the total cumulative prone time varied considerably across and within studies, which may have contributed to heterogeneity in the results.

**Table 3:** Summary of findings

Outcomes	Study design	Risk-of-bias	No. of patients		Effect Relative (95% CI)	Certainty
			Prolonged (≥ 24 h)	Standard (< 24 h)		
*Primary outcomes*						
Short-term mortality	RCT	Not serious	20/56 (35.7%)	18/56 (32.1%)	RR 1.07 (0.03 to 44.06)	⨁⨁◯◯ Low
Long-term mortality	RCT + NRSI	Not serious	145/421 (34.4%)	94/216 (43.5%)	HR 0.72 (0.41 to 1.25)	⨁⨁◯◯ Low
*Secondary outcomes*						
Total time in prone position	RCT +	Not serious	56	56	SMD 0.79 SD higher (2.65 lower to 4.24 higher)	⨁◯◯◯ Very low
Change in PaO₂/FiO₂	RCT	Not serious	56	56	SMD 0.24 SD lower (2.26 lower to 1.79 higher)	⨁⨁◯◯ Low
Pressure injuries	RCT	Not serious	26/56 (46.4%)	7/56 (12.5%)	RR 1.79 (0.00 to 1000 more)	⨁◯◯◯ Very low
Loss-of-catheters or endotracheal tube	RCT + NRSI	Not serious	11/239 (4.6%)	10/188 (5.3%)	RR 0.86 (0.18 to 4.13)	⨁◯◯◯ Very low

CI: confidence interval; HR: hazard ratio; NRSI: non-randomized trial of interventions; PaO₂/FiO₂ ratio: ratio of arterial partial pressure of oxygen to fractional inspired oxygen; RCT: randomized controlled trial; RR: risk ratio; SMD: standardized mean difference

Prone positioning is a cornerstone therapy for patients with moderate to severe ARDS and one of the few interventions proven to improve survival in randomized trials. Early studies employing relatively short prone sessions reported no clinical benefit, whereas subsequent trials demonstrated that sessions of at least 16 h, in combination with lung-protective ventilation strategies, significantly reduce mortality. Nevertheless, the optimal duration of prone positioning remains uncertain. Prolongation of sessions may help sustain alveolar recruitment and mitigate ventilator-induced lung injury, potentially improving outcomes, but it may also increase the need for deeper sedation and the risk of complications such as pressure injuries, thereby offsetting potential benefits.

A recent meta-analysis by Kang et al. [40] reported improved oxygenation with PPP in mechanically ventilated COVID-19 patients with ARDS, but did not demonstrate a survival benefit. PPP was associated with a higher incidence of pressure injuries. However, several methodological concerns have been raised that may limit the reliability of these findings [41], including the inclusion of non-randomized studies with unadjusted outcome data, errors in data extraction, and misclassification of treatment groups. In our review, we sought to address these limitations by clearly distinguishing between randomized and non-randomized evidence, applying rigorous risk of bias assessments, and systematically evaluating the certainty of evidence. Given the limited number and small sample sizes of available RCTs, we pooled them with observational studies to increase statistical power and provide a comprehensive overview of the current evidence to inform clinical decision-making. We acknowledge that this approach may introduce confounding by indication, which we sought to address through our analytical strategy. This included the application of Mantel-Haenszel adjustment across all syntheses and a clear differentiation between NRSIs that provided robust adjustment for baseline confounding and those that did not. To capture the full evidence base, we reported pooled analyses including all eligible studies. To ensure the robustness of findings, additional analyses restricted to studies assessed as low to moderate risk of bias were conducted and presented alongside full dataset analyses to enable direct comparison. To enable a differentiated appraisal according to study design and quality, results were also reported separately for RCTs and NRSI as sensitivity analyses.

For overall mortality, the point estimate from the sensitivity analysis, which included all eligible studies regardless of risk-of-bias, favored prolonged prone positioning. However, this analysis relied on unadjusted raw data and included studies with a high risk of bias, warranting cautious interpretation of the results. Similarly, the analysis of pressure injuries encompassing all eligible studies, suggested a trend toward increased risk in the prolonged prone group. Nonetheless, given the inclusion of numerous studies with high risk of bias and unadjusted data, all analyses that were not restricted to studies with low or moderate risk of bias should be interpreted with considerable caution due to its limited reliability.

A key challenge in interpreting the available evidence is the lack of a standardized definition for “prolonged” prone positioning. While we defined prolonged sessions as lasting more than 24 h, other studies have used different thresholds, making direct comparisons difficult. In our primary analysis, we included studies that explicitly compared prone positioning durations of less than 24 h versus more than 23 h per session, as defined in their methods sections.

The absence of a validated definition of PPP is further compounded by inconsistent or insufficiently specified criteria for treatment group allocation across the observational studies. Most observational studies categorized patients according to the duration of the initial prone session, while subsequent session durations were frequently underreported. Across the observational studies, crossover between groups occurred, particularly during subsequent prone positioning sessions, thereby limiting the validity of direct comparisons. Moreover, criteria for discontinuation of prone positioning were either not specified or heterogeneously defined (Table S4). Such variability in study design and outcome reporting substantially limits the robustness of pooled analyses and highlights the need for standardized definitions and prospective trials to allow more robust conclusions.

It remains uncertain whether patient outcomes are primarily influenced by the duration of individual prone positioning sessions or by the cumulative exposure to prone positioning over the entire treatment course. As ventilation in the prone position is considered more lung-protective than in the supine position, the overall proportion of time spent prone may be an important determinant of clinical benefit. However, longer cumulative prone positioning may also increase risks, including the need for deeper sedation and pressure-related injuries. Notably, total prone time differed substantially across included studies, which likely contributed to heterogeneity in the reported findings. To reduce heterogeneity and allow a clearer assessment of benefits and risks, future studies should consistently report and ideally standardize cumulative prone exposure, along with predefined criteria for discontinuation. This approach would enable a more accurate evaluation of its effect on clinically relevant outcomes.

In addition to methodological issues, population characteristics warrant consideration. Our systematic review predominantly identified studies evaluating patients with ARDS secondary to SARS-CoV-2 infection that directly compared standard versus prolonged prone positioning. Although some investigations have examined prolonged prone positioning in non-COVID ARDS, these did not include clearly defined comparator groups for standard and prolonged proning [13, 22, 42, 43]. While COVID- and non-COVID ARDS share important pathophysiological features, it remains uncertain whether evidence derived from COVID-19 populations can be generalized to non-COVID ARDS. Moreover, changes in clinical practice during the pandemic, such as resource constraints and staff shortages, may have contributed to longer prone sessions, thereby potentially limiting the applicability of these findings beyond the COVID-19 context.

This meta-analysis has several important limitations. A major concern is the potential for confounding by indication inherent to the observational studies evaluating prolonged prone positioning. Because prone positioning typically leads to improved oxygenation, patients with more severe or persistent hypoxemia were more likely to be maintained in the prone position for extended durations. As a result, these individuals may represent a subgroup with an inherently worse baseline prognosis, thereby biasing the association between prolonged proning and clinical outcomes. To address this, we conducted analyses limited to studies assessed as having low to moderate risk of bias to ensure the reliability of the findings by focusing on the best available evidence. Another major limitation of this review is the variability in the threshold definitions for prolonged prone positioning across included studies, ranging from 24 to 74 h, which reduces comparability and complicates interpretation. Another key limitation is the limited number of eligible studies comparing prolonged and standard prone durations. Consequently, subgroup analyses were not performed due to insufficient data to support reliable conclusions. Additionally, restricting inclusion to studies published in English and German may have introduced language bias.

This systematic review also possesses several strengths. A key strength of our systematic review lies in its comprehensive and methodologically rigorous approach, strictly adhering to PRISMA guidelines, transparent reporting of methodology, and with prospective protocol registration in PROSPERO to ensure transparency and reduce risk of bias. We applied a thorough risk-of-bias assessment and evaluated the certainty of evidence using the GRADE framework, enhancing the reliability and interpretability of our findings. By synthesizing data from both randomized controlled trials and observational studies, this review addresses a critical evidence gap regarding prolonged prone positioning in ARDS, directly informing clinical decision-making. Our findings support guideline-based [44] prone positioning (≥ 16 h per session) and discourage routine use of prolonged sessions outside of clinical trials.

## Conclusion

Our findings align with current international guidelines recommending prone positioning for at least 16 h per session, but do not support routine use of prolonged prone sessions outside clinical trials. Consistent with prior meta-analyses and large cohort studies, we found no clear evidence to support further extension beyond this duration. Future research should prioritize randomized controlled trials employing standardized definitions and protocols, with systematic reporting of both individual session length and cumulative prone time. Such studies are essential to determine whether particular patient subgroups may derive benefit from tailored proning strategies. Until such evidence emerges, adherence to current guideline recommendations remains the most appropriate clinical approach.

## Supplementary Information

Supplementary Material 1

Supplementary Material 2

Supplementary Material 3

## Data Availability

The following materials are not publicly available, but can be obtained from the corresponding author upon reasonable request: template data collection forms; data extracted from included studies; data used for all analyses; analytic code; and other materials used in the review.

## Abbreviations

aHR: Adjusted hazard ratio
ARDS: Acute respiratory distress syndrome
BMI: Body mass index
COVID: Corona-virus-disease of 2019
IQR: Interquartile range (quartile 1–quartile 3)
HR: Hazard ratio
NRSI: Non-randomized study of intervention
PaO₂/FiO₂: Ratio of arterial oxygen partial pressure to fractional inspired oxygen
PP: Prone position
PPP: Prolonged prone position
RCT: Randomized controlled trial
ROB-2: Revised tool to assess risk of bias in randomized trials
ROBINS-I: Risk of bias in non-randomized studies – of interventions
ROB-ME: Tool for assessing risk of bias due to missing evidence
SARS-CoV-2: Severe acute respiratory syndrome coronavirus 2
SD: Standard deviation
SMD: Standardized mean difference
VILI: Ventilator-induced lung injury
