# Supplementary material for: Effectiveness and safety of prolonged prone positioning in adult patients with acute respiratory distress syndrome (ARDS): a systematic review and meta-analysis
Source: Crit Care. 2025 Nov 6;29:475. doi: 10.1186/s13054-025-05712-0 (PMC12590638; doi:10.1186/s13054-025-05712-0)
Supplement: Supplementary file 3 — Supplementary Material 3 [file 13054_2025_5712_MOESM3_ESM.docx]

**Online Data Supplement**

**Effectiveness and safety of prolonged prone positioning in adult patients with acute respiratory distress syndrome (ARDS): a systematic review and meta-analysis**

Carolin Jung, Hans-Joerg Gillmann, Thomas Stueber

**Table of Contents page**

1. **Search strategy 2**
2. **Analytical assumptions 3**
3. **Table S1. List of excluded studies 4-5**
4. **Table S2. List of ongoing studies 6**
5. **Table S3. Detailed GRADE assessment 7**
6. **Table S4. Detailed Characteristics of Outcome Measures 8
   Across Included Studies**
7. **Table S5. ROB-ME Assessment 9**
8. **Table S6A-C. Detailed ROB-2 Assessment 10-12**
9. **Figures S1-2. Sensitivity Analyses 13-14**
10. **References 14**

*Search strategy*

We systematically searched the following electronic databases: MEDLINE, EMBASE, and the Cochrane Central Register of Controlled Trials (CENTRAL). In addition, we screened the following trial registries for ongoing or unpublished studies: ClinicalTrials.gov, the ISRCTN registry (http://isrctn.com), the World Health Organization International Clinical Trials Registry Platform (ICTRP) (www.who.int/clinical-trials-registry-platform), and the Cochrane COVID-19 Study Register (<https://covid-19.cochrane.org>).

The core search terms were:

*("acute respiratory distress syndrome" OR "ARDS" OR "acute lung injury" OR "ALI" OR "respiratory failure" OR "COVID") AND ("pron*")*

This search syntax was applied consistently across all databases, except for ClinicalTrials.gov and ISRCTN, where it was adapted to meet platform-specific requirements:

*("acute respiratory distress syndrome" OR ARDS OR "acute lung injury" OR ALI OR "respiratory failure" OR COVID) AND (prone OR pronation OR proning)*

*Analytical assumptions*

1. If change-from-baseline standard deviations were not reported, they were imputed following the guidance from the Cochrane Handbook (version 6.5) with the following formula (1):


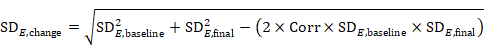


In cases where the correlation coefficient between baseline and post-intervention measurements was unavailable, a conservative value of r=0.5 was assumed, reflecting the uncontrolled nature of the included trials and the considerable heterogeneity observed among reported standard deviations. Such an imputation was necessary for PaO₂/FiO₂ change in the following studies: Rezoagli et al., 2021; Page et al., 2022; Karlis et al., 2023.

2. In the RCT by Saez de la Fuente, hazard ratios (HR) were not reported; therefore, we approximated them indirectly from the Kaplan-Meier curve for the analysis of long-term mortality (2). Tierney JF, Burdett S, Fisher DJ. Practical methods for incorporating summary time-to-event data into meta-analysis: updated guidance. Syst Rev. 2025;14(1):84.).

3. For the meta-analyses of total prone time and changes in PaO₂/FiO₂, when studies reported medians and interquartile ranges (IQR) instead of means and standard deviations (SD), means and SDs were estimated using the Box-Cox method (implemented with the *estmeansd* package in R. The estimated values were then used for quantitative synthesis. (McGrath S, Zhao X, Steel R, Benedetti A. estmeansd: Estimating the Sample Mean and Standard Deviation from Commonly Reported Quantiles in Meta-Analysis. R package version 1.0.1; 2025.)

**Table S1. List of excluded studies**

| **Study** | **Publication date** | **Design** | **Study setting** | **Population** | **Duration of PP /Intervention** | **Duration of PP /Control** | **Case number (Intervention vs. Control)** | **Reason for Exclusion** |
| --- | --- | --- | --- | --- | --- | --- | --- | --- |
| Romero  et al. | 2009 | Prospective NRSI |  | Severe ARDS | 55 ± 7 hours | NI | 15 | Lack of control group. |
| Kimmoun et al. | 2015 | Retrospective NRSI |  | Severe ARDS AND ECMO | 24 hours | NI | 17 | Lack of control group. |
| Ruste  et al. | 2018 | Retrospective NRSI | Single-center | ARDS | NI | 16 ± 3 | 197 | Lack of control group. |
| Jochmans et al. | 2020 | Prospective NRSI | Single-center | All patients with prone position ( 95% hatten ARDS) | NI | 21.5 ± 5 | 103 | Lack of control group. |
| Carsetti  et al. | 2020 | Retrospective NRSI | Single-center |  | 16 | 36 (33.5 - 39) | 10 (6 mixed, 3 prolonged, 1 standard) | Case series with less than 5 patients in each arm |
| Ibarra  et al. | 2020 | Retrospective NRSI | Single-center | Moderate-to-severe ARDS, COVID-19 | **NA** | **NA** | 74 | Different comparator (patients with vs. without positioning-related injuries). |
| Cherukuri et al. | 2021 | Retrospective NRSI | Single-center | ARDS, COVID-19 | **NA** | **NA** | 212 | Different comparator (survivors vs. non-survivors); published only as a conference abstract. |
| Douglas et al. | 2021 | Retrospective NRSI | Single-center | ARDS, COVID-19 | 4.9 (2.1-10) days | NA | 61 | Lack of control group. |
| Parker  et al. | 2021 | Retrospective NRSI | Single-center | ARDS, COVID-19 | > 39 hours | „standard“ | 20 | No definitive duration of the individual prone session given. |
| Shearer  et al. | 2021 | Retrospective NRSI | Single-center | ARDS, COVID-19 | NI | NI | 143 | Different comparator (patients with vs. without positioning related injuries); no definitive duration of the individual prone sessions given. |
| Cornejo  et al. | 2022 | Retrospective NRSI | Single-center | Moderate-to-severe ARDS, COVID-19 | 2-3 days, vs. 4-5 days, vs. > 5 days | NI | 417 (191 vs. 128 vs. 98) | All three groups were prolonged prone, without a standard control. |
| Lee  et al. | 2022 | Retrospective NRSI | Single-center | ARDS, non-COVID-19 | 66.1 (44.4 - 84-5) | NI | 116 | Lack of control group. |
| Hochberg et al. | 2022 | Retrospective NRSI | Multi-center | ARDS, COVID-19 and Non-Covid-19 | 43 (28.3-60.3; COVID-19) vs. 28 (21.5-42; historical) | NA | 512 | Different comparator (COVID-19 ARDS vs. historical ARDS). |
| Walter  et al. | 2022 | Retrospective NRSI | Single-center |  | 39 (34-42) hours* | NI | 81 | Lack of control group. |
| Miguel-Balsa  et al. | 2023 | Retrospective NRSI | Single-center |  | < 24 hours | > 24 hours | 51 | No definitive duration of prone session given (only information given: > 24 vs. < 24 hours) |
| Estrella-Alonso  et al. | 2024 | Retrospective NRSI, Adjustment for baseline confounding | Single-center | Moderate-to-severe ARDS, COVID-19 | 44 (37 - 47) | 24 (21 - 26) | 156 (82 vs. 76, both cohorts ≥ 24 h prone) | Both groups were prolonged prone, without a standard control. |
| Gonzalez-Castro  et al. | 2024 | Retrospective NRSI | Single-center | ARDS, COVID-19 | NI | 14 (95% CI 10-16) vs. 19 (18 - 20) vs. 22 (21-24) hours | 271 | All groups were standard prone. |
| Makhija  et al. | 2025 | Prospective NRSI | Single-center | Severe ARDS (according to Berlin Criteria) | 36 (32-40) hours | NI | 72 | Lack of control group; published only as conference abstract. |
| Yan  et al. | 2025 | Retrospective NRSI, Propensity Score Matching | Single-center | ARDS (mild to severe) | 11 (8.13 - 14.12) | 20.1 (17.8 - 225.6) | 162 (81 vs. 81) | Both groups were standard prone. |

Data are presented as mean ± standard deviation or median (Q1–Q3). When two groups are compared (vs.), values for the prolonged group are listed first, followed by those for the standard group. If NRSI had adjustment for baseline confounding it was specified in column “Design”.

*ARDS: Acute respiratory distress syndrome; NA: Not applicable due to different comparator; NI: No information available; NRSI: non-randomized study of interventions; RCT: randomized controlled trial;*

| **List of ongoing studies** | **1** | **2** | **3** |
| --- | --- | --- | --- |
| Study name | Effect of Continuous Prolonged Prone Position Versus Intermittent Daily Prone Position in ARDS (ePRONE) | Clinical Impact of Different Duration Prone Postition Treatment for Patients With ARDS. | Efficacy and safety of long-term prone ventilation in patients with critically ill COVID-19 pneumonia: a single-center, prospective, randomized controlled study |
| Design | RCT | RCT | RCT |
| Estimated no. of participants | NI | NI | NI |
| Inclusion criteria | Age ≥ 18 years Endotracheal intubation and mechanical ventilation for less than 72 hours Moderate-severe ARDS defined as:  Within 1 week of a known clinical insult or new or worsening respiratory symptoms Bilateral infiltrates not fully explained by effusions, lobar/lung collapse, or nodules Respiratory failure not fully explained by cardiac failure or fluid overload PaO2/FiO2 < 150 mmHg in supine position Prone positioning has been indicated by the attending physician, OR has already been initiated within the last 16 hours | Patient above 20 year-old with diagnosis of severe ARDS under protective lung ventilation (tidal volume 4-8 ml/kg, plateau pressure < 30cm H2O、PaO2/FiO2 < 150 mmHg、PEEP ≥ 5 cmH2O、FiO2 > 60%). | Inclusion criteria: (1) Meet the diagnostic criteria of the tenth edition of the diagnosis and treatment protocol "critical COVID-19"; (2) Age 18-85 years old (3) Patients undergoing mechanical ventilation; |
| Exclusion criteria | Contraindications for prone positioning such as intracranial pressure > 20 mmHg, massive hemoptysis, recent tracheal surgery or sternotomy or abdominal surgery with an open wound, recent facial trauma or facial surgery, unstable spine, femur, or pelvic fractures, or a single anterior chest tube with air leaks Patient on extracorporeal membrane oxygenation (ECMO) before randomization Chronic respiratory failure requiring oxygen therapy or non-invasive ventilation (NIV) Known pregnancy Anticipating withdrawal of life support or shift to palliative care | Patients were not intubated and mechanically ventilated or contraindication for prone position ventilation (intracranial hemorrhage, massive hemoptysis, unstable hemodynamic status, recent pacemaker implantation, severe facial laceration, open abdominal wound, spine, femur or pelvis fracture or pregnancy). | Exclusion criteria: (1) severe intra-abdominal pressure increase (2) intracranial pressure increase (3) Recent history of facial, chest and abdominal, fracture, spine and other operations; (4) Confirmed venous thromboembolism, and anticoagulation treatment less than 2 days; (5) Hemodynamic instability; (6) pregnancy status; (7) Research objects deemed unsuitable by researchers. |
| Intervention | PP at least 48 hours AND until PaO2/FiO2 ratio is ≥ 200 mmHg, but no longer than 120 hours. | PP 24 hours | PP > 24 hours |
| Comparator | PP 16-24 hours | PP 16 hours | Standard PP duration |
| Outcome | Mortality | Oxygenation | Oxygenation index |
| Starting date | 2025-04-07 | 2020 (Completion 2023-07-21) | 2023-07-10 |
| Author contact information | Alejandro Bruhn, MD, PhD, Phone Number: +56223543292, Email: abruhn@uc.cl | Chien Wie Hsu, Kaohsiung Veterans General Hospital; Kaohsiung, Taiwan, 813 | Yinchuan Li ; Phone Number: +86 136 8194 5425;  Email: yingchuan.li@sjtu.edu.cn |
| Notes | NCT06854627 | [NCT04391387](https://clinicaltrials.gov/study/NCT04391387) | ChiCTR2300073402 |

**Table S2. List of ongoing studies**

**Table S3. Detailed GRADE Assessment**

**
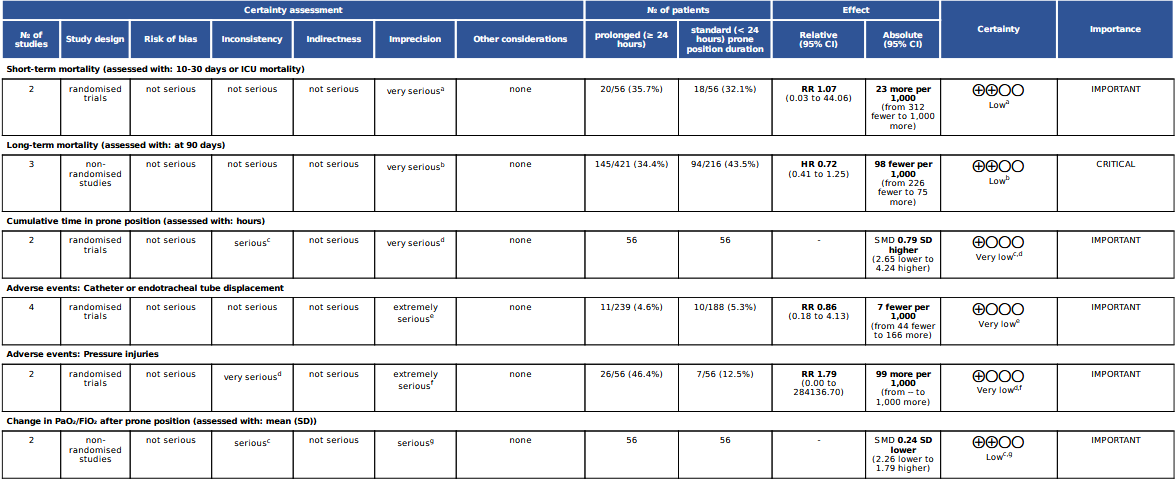
***CI: confidence interval; HR: hazard ratio; NRSI: non-randomized study of interventions; PaO₂/FiO₂: ratio of arterial oxygen partial pressure to fractional inspired oxygen; RR: risk ratio; SMD: standardized mean difference; RCT: randomized controlled trial*

| **Study** | **Design** | **Capture of PaO2/FiO2 ratio after Initiation of PP** | **Number of position changes** | **Lung-protective ventilation** | **Use of neuromuscular blockade** | **Rescue therapy: ECMO** | **Have clear criteria for stopping of prone position**  **been defined?** | **Crossover** |
| --- | --- | --- | --- | --- | --- | --- | --- | --- |
| Rezoagli et al., 2021 | NRSI | Within 2-6 hours after re-supination | 2 (2-4) vs.  4 (2-5) | Yes | NI | NI | NI | NI |
| Shinner et al., 2021 | NRSI | NI | NI | NI | NI | NI | NI | NI |
| Lucchini et al.  2021 | NRSI | NI | 3 (2-4) vs.  2 (1-4) | NI | NI | 7 vs. 6 | No | Yes |
| Hafez et al.,  2022 | RCT | 1 hour after re-supination | NA | Yes | NI | NI | Yes (Prolonged: 24 hours; Standard: 16 hours) | NI |
| Page et al.,  2022 | RCT | 96 hours after begin of prone therapy | 4.6 ± 1.6 vs.  5.7 ± 2.6 | Yes | NI | 0 vs. 1 | Yes (Prolonged: 24 hours; Standard: 16 hours) | NI |
| Karlis et al.,  2023 | NRSI | 4 hours after re-supination | 1 (1-2) vs.  2 (1-3) | Yes | NI | No | No | NI |
| Okin et al.,  2023 | NRSI | Within 6 hours after pronation | 1 (1-2) vs.  3 (1-4) | Yes | 27.4 vs. 43.6% received NMBA | 4 vs. 10 | No | Yes |
| Hochberg et al.,  2024 | NRSI | Within 12-16 hours of proning | 2 (1-3) vs.  2 (2-4) | Yes | 50 vs. 57% received NMBA | No (Predefined exclusion criterion) | No | Yes |
| Saez de la Fuente et al., 2024 | RCT | 1 hour after re-supination | 2 (1-3) vs.  2 (1-3) | Yes | **Duration:**  12.5 (5.8-20) vs. 5 (2-14.5) days | 2 vs. 0 | Yes (Prolonged: after 48 hours; Standard: any time after 16h if there was an improvement in oxygenation; Both groups: whenever a life-threatening complication occurred) | NI |

**Table S4. Detailed Characteristics of Outcome Measures Across Included Studies**

Data are presented as mean ± standard deviation or median (Q1–Q3). When two groups are compared (vs.), values for the prolonged group are listed first, followed by those for the standard group.

*ECMO: extracorporeal membrane oxygenation; NI: No information available; NMBA: neuromuscular blockade; NRSI: Non-randomized study of interventions; RCT: randomized controlled trial;*

**Table S5. ROB-ME assessment**


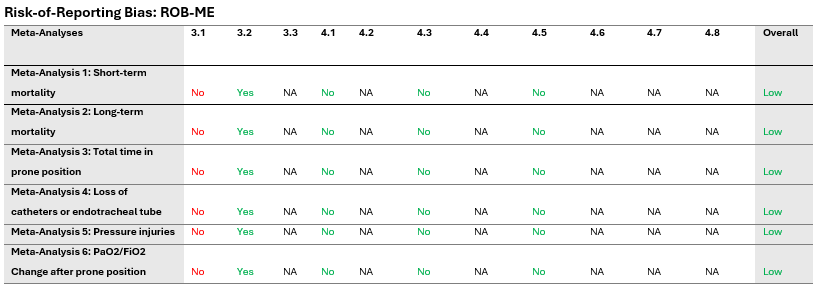


NA: This item was considered not applicable according to the ROB-ME algorithm, since the responses to prior questions precluded its assessment.

**Table S6A: Detailed ROB-2 Assessment for Saez de la Fuente et al., 2024**

**Table S6B: Detailed ROB-2 Assessment for Page et al., 2022**

**Table S6C: Detailed ROB-2 Assessment for Hafez et al., 2022**

**Fig. S1A: Sensitivity Analysis: Pooled Mortality**


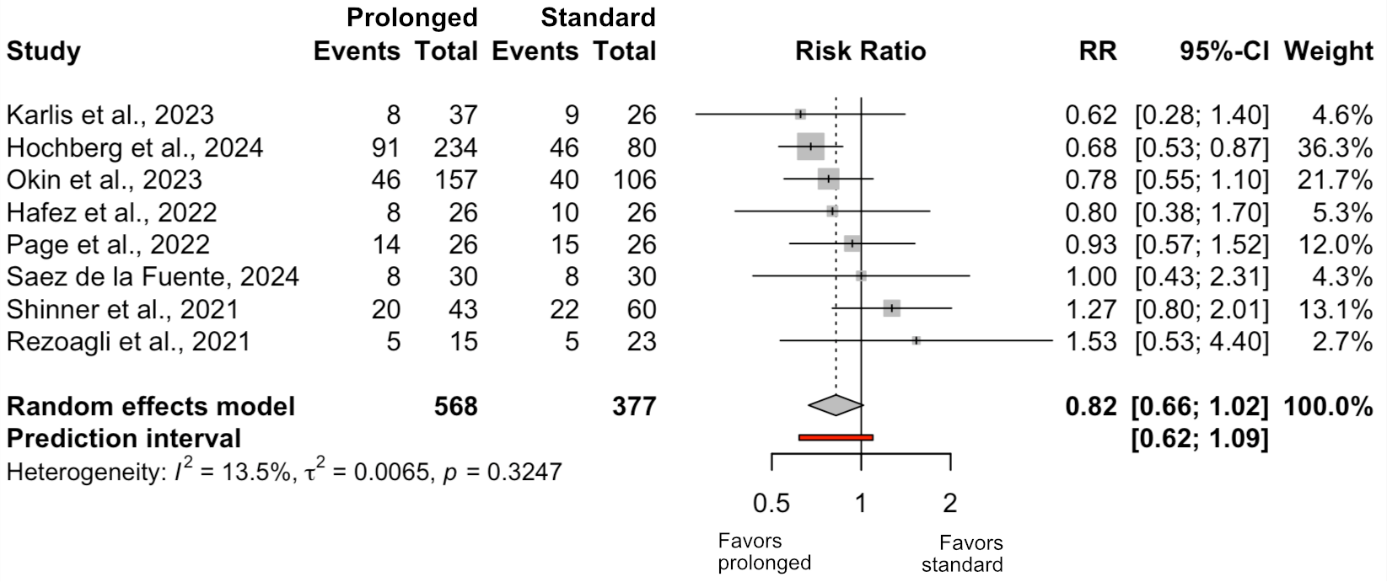


The included studies reported the most recently available data on mortality, with varying follow-up periods: 28-day mortality in Karlis et al., 30-day mortality in Page et al., ICU mortality in Rezoagli et al., hospital mortality in Hafez et al., and 90-day mortality in Hochberg, Okin, and Saez de la Fuente et al. Shinner did not specify the time point for mortality assessment. All eligible studies were incorporated into this sensitivity analysis, irrespective of their risk of bias.

**Fig. S1B: Sensitivity Analysis: Short-term mortality – only RCTs**


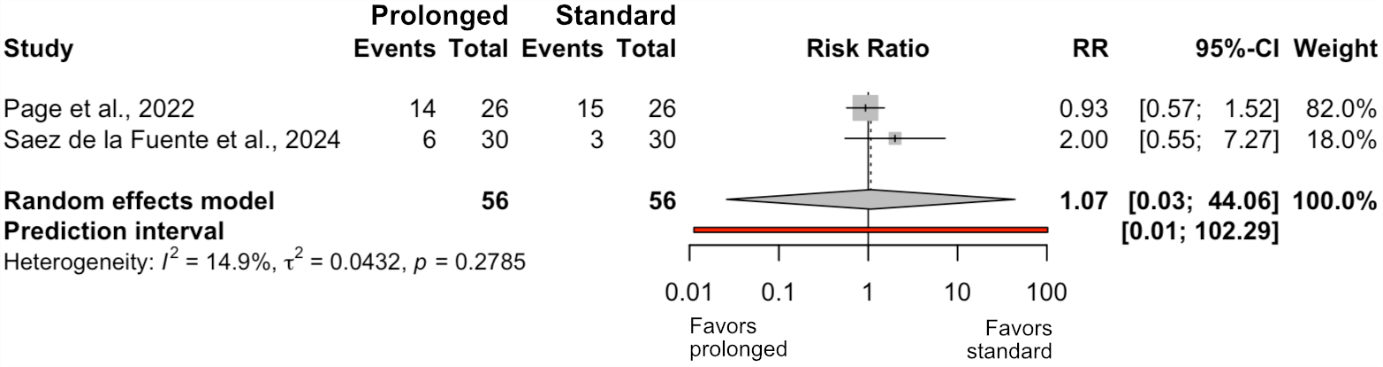


**Fig. S1C: Sensitivity Analysis: Short-term mortality – only RCTs, regardless of risk-of-bias**

**
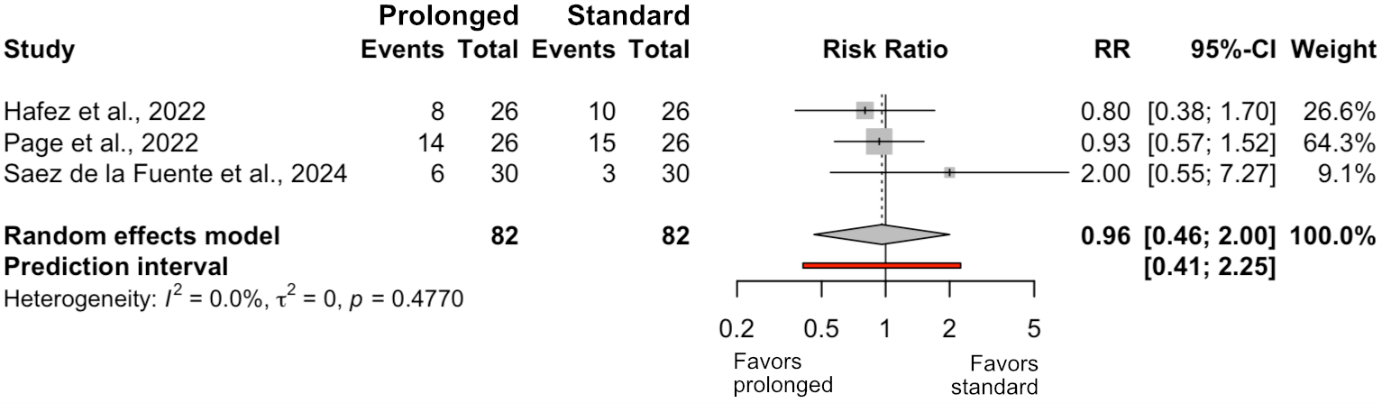
**

**Fig. S1D: Sensitivity analysis: Long-term mortality: Only RCTs**

**
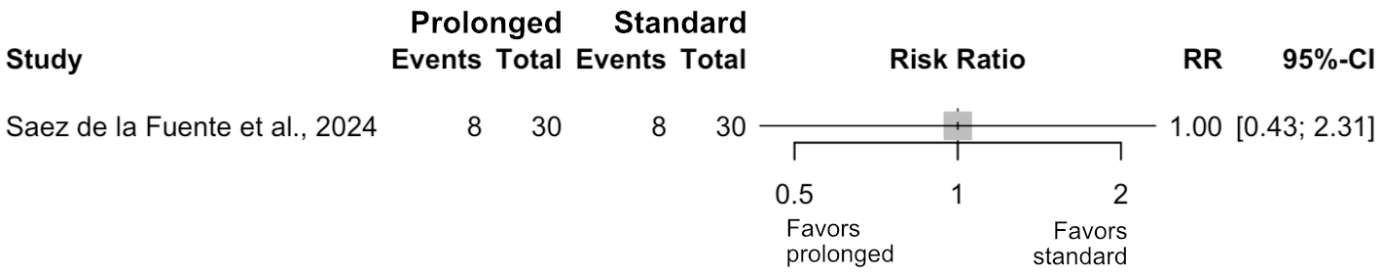
**

**Fig. S1E: Sensitivity analysis: Long-term mortality: Only NRSIs**

**
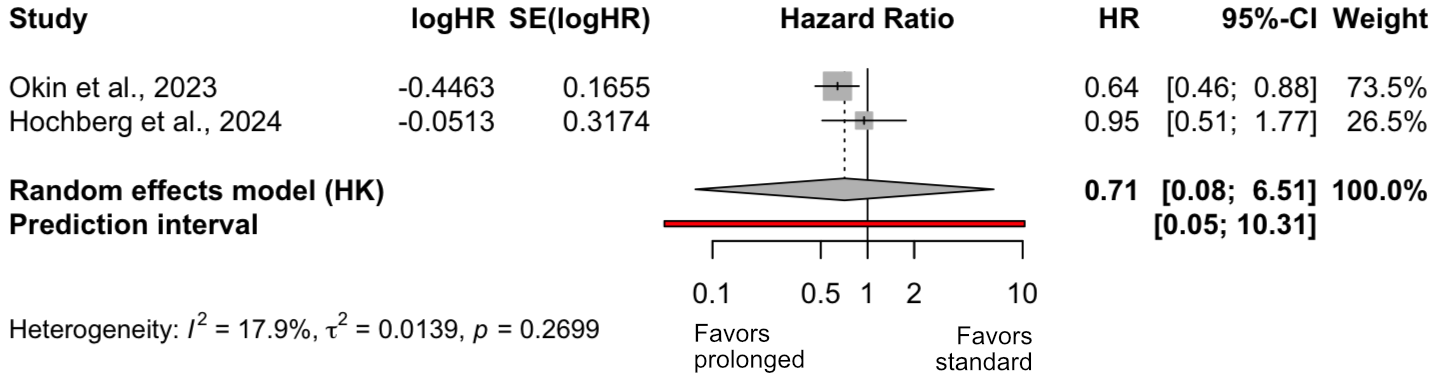
**

**Fig. S2: Sensitivity Analysis: Incidence of loss-of-catheters or endotracheal tube: RCT only**


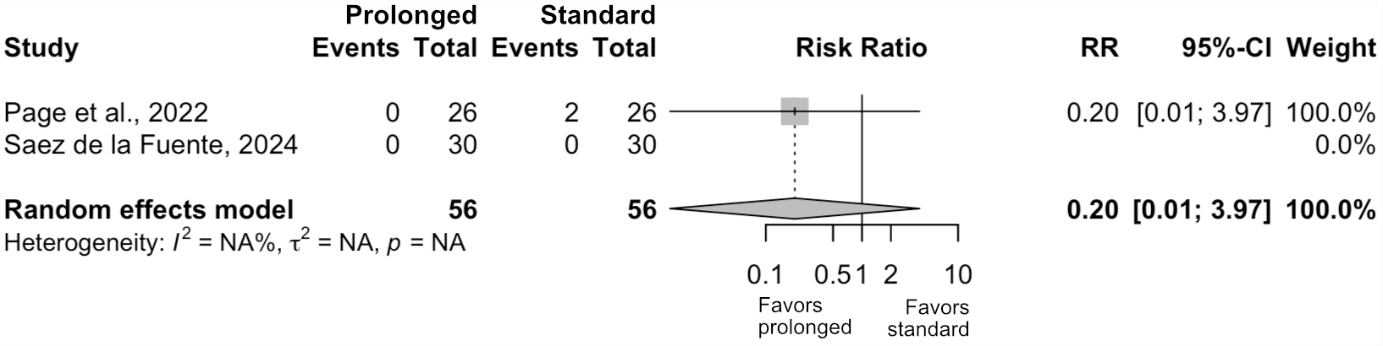


**References**

1. Higgins JPT LT, Deeks JJ (editors). Chapter 6: Choosing effect measures and computing estimates of effect Cochrane; 2024 [Available from: <https://www.cochrane.org/authors/handbooks-and-manuals/handbook/current/chapter-06>.
